# Does Hallux Valgus Impair Medial Forefoot Loading? A Meta‐Analysis of Plantar Pressure Distribution

**DOI:** 10.1002/jfa2.70073

**Published:** 2025-08-11

**Authors:** Duo Wai‐Chi Wong, Esther Man‐Wai Chow, Lucci Lugee Liyeung, Jiao Wang, Toby Chi‐To Mak, James Chung‐Wai Cheung, Ming Ni, Aaron Kam‐Lun Leung

**Affiliations:** ^1^ Department of Biomedical Engineering Faculty of Engineering The Hong Kong Polytechnic University Hong Kong China; ^2^ Department of Orthopaedics and Traumatology Prince of Wales Hospital Hong Kong China; ^3^ Department of Rehabilitation Sciences Faculty of Health and Social Sciences The Hong Kong Polytechnic University Hong Kong China; ^4^ Department of Orthopaedics Ruijin Hospital School of Medicine Shanghai Jiaotong University Shanghai China; ^5^ Laboratory of Prevention and Treatment of Bone and Joint Diseases Ruijin Hospital School of Medicine Shanghai Institute of Traumatology and Orthopaedics Shanghai Jiaotong University Shanghai China

**Keywords:** foot deformity, forefoot biomechanics, hallux abducto valgus, meta‐analysis, metatarsus primus varus, pedobarography

## Abstract

**Background:**

Hallux valgus is a common foot deformity that may alter plantar pressure distribution. This study aimed to determine whether hallux valgus consistently reduces medial forefoot (hallux and medial metatarsal) load and impulse through a meta‐analysis of plantar pressure data and increases central metatarsal loading.

**Methods:**

A systematic review and meta‐analysis were conducted following PRISMA guidelines. PubMed, Web of Science, Scopus, CINAHL, and Embase were searched for studies comparing plantar pressure distribution between hallux valgus and non‐hallux valgus feet. Random‐effects meta‐analyses were performed using standardized mean differences (SMD) for load (force and pressure) and impulse (force and pressure‐time integrals) measures in hallux, medial metatarsal, and central metatarsal regions. Risk of bias was assessed using the JBI critical appraisal checklist for analytical cross‐sectional studies.

**Results:**

Twenty‐one studies (3911 participants and 6407 feet) were included. In‐shoe measurements showed significant reductions in hallux loading (force SMD: −0.78 [95% CI: −1.08, −0.49]; force‐time integral SMD: −0.39 [−0.71, −0.07]). Platform‐based measurements yielded inconsistent results. No conclusive evidence was found for reduced medial metatarsal loading or increased central metatarsal loading. Measurement modality significantly influenced results, with platform‐based systems generally showing higher heterogeneity than in‐shoe sensors. Nine (43%) and 12 studies (57%) had a high risk of bias on statistical analysis and confounder management, respectively.

**Conclusions:**

Hallux valgus is associated with reduced hallux loading in in‐shoe measurements, but evidence for load redistribution to other forefoot regions is inconclusive. The choice of measurement modality significantly impacts results, highlighting the need for standardized assessment protocols in hallux valgus research.

**Trial Registration:**

This study was registered in PROSPERO with the reference number CRD42024574195

## 1 Introduction

Hallux valgus (hallux abducto valgus), commonly known as a bunion, is a prevalent foot deformity characterized by the lateral deviation of the hallux and medial deviation of the first metatarsal. Although bunion refers to the physical prominence at the first metatarsophalangeal joint, hallux valgus specifically describes the angular deformity of the hallux. The condition affects approximately one‐third of the older adults, with its prevalence increasing with age and being higher among females [1]. The impact of hallux valgus extends beyond cosmetic concerns, often resulting in foot pain, functional limitations, an increased risk of falls, secondary foot problems, and a reduced quality of life [2, 3]. Understanding the biomechanical effects of hallux valgus is crucial for developing effective treatment strategies and improving outcomes [4].

**FIGURE 1:**
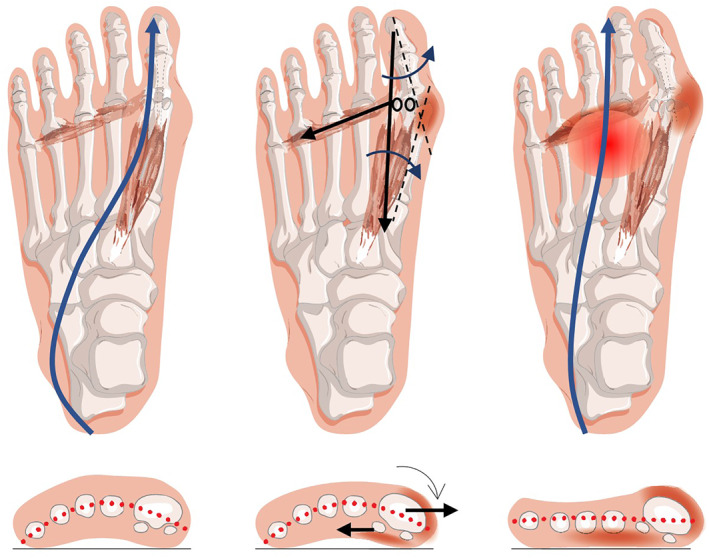
Progression of hallux valgus, its impact on plantar load distribution, and transfer metatarsalgia [4] under the Creative Commons Attribution license.

Theoretically, hallux valgus alters the plantar pressure distribution and load‐bearing capability of the forefoot due to its structural changes [5]. There is typically a decrease in loading under the hallux and a compensatory increase in pressure under other regions, leading to other foot problems, such as metatarsalgia [6]. The biomechanical deterioration in hallux valgus is initiated by progressive first ray instability, where ligament laxity permits medial displacement of the first metatarsal head (metatarsus primus varus) [7, 8]. This medial shift of the first metatarsal head, rather than true lateral migration of the sesamoids, creates the appearance of sesamoids being positioned laterally relative to the mechanical axis. This process generates a destabilizing valgus moment at the first metatarsophalangeal joint [9] and disrupts two critical load‐bearing mechanisms: the windlass mechanism, which relies on an intact plantar aponeurosis to stabilize the medial column during propulsion [10], and the physiological center‐of‐pressure trajectory that normally progresses medially during push‐off [11] (Figure 1). As deformity progresses, lateral bowstringing of the hallucis tendon transforms it from a stabilizer into a deforming force [12], and erosion of the crista ridges further undermines the hallux's load‐bearing architecture [9]. These anatomical failures initiate a self‐perpetuating cycle, where medial column instability leads to compensatory lateral weight shifts, further destabilizing the first ray [13]. Additionally, in a normal foot, the first ray actively plantarflexes during terminal stance, bearing significant load and protecting the second metatarsal head from excessive pressure. However, the medially deviated first ray does not flex properly, known as the first ray insufficiency, which prevents natural off‐loading of the second and third metatarsal heads [14]. This is clinically evident as plantar callosities, hyperkeratosis, and metatarsalgia [15, 16], which often trouble patients more than the bunion itself.

Clinical studies have shown that hallux valgus feet often exhibit reduced pressure and load under the hallux, with negative correlations between hallux valgus angles [5]. This decreased loading of the hallux is accompanied by increased pressure and force under the lesser toes and the second and third metatarsal heads [17]. However, it is important to note that there are conflicting results in the literature regarding plantar pressure distribution in hallux valgus [6, 17, 18].

Additionally, recent research has raised questions about the biomechanical effectiveness of surgical interventions for hallux valgus. Although some surgical designs focus on improving biomechanical functions, particularly intermetatarsal stability [19, 20], a meta‐analysis suggested that surgeries, in general, might not only be ineffective but could potentially worsen plantar pressure distribution [4]. However, this conclusion assumed that hallux valgus inherently reduces medial forefoot loading, an assumption challenged by conflicting evidence in the literature. The inconsistency in reported plantar pressure patterns raises concerns about the validity of using plantar pressure assessment for clinical decision‐making and evaluating hallux valgus interventions.

Therefore, this study aims to focus on a quantitative synthesis (meta‐analysis) to determine whether hallux valgus consistently reduces medial forefoot loading or pressure. We hypothesized that the load and impulse under the hallux and medial metatarsal regions would decrease due to impaired load‐carrying capacity, whereas the load and impulse under the central metatarsal region would increase as a compensatory mechanism, potentially leading to transfer metatarsalgia. This paper adhered to the reporting guidelines of PRISMA and was registered in PROSPERO with the reference number CRD42024574195.

## 2 Methods

### 2.1 Eligibility Criteria

This study aimed to compare plantar pressure distribution between hallux valgus and non‐hallux valgus. We included studies that were either cross‐sectional, comparing hallux valgus and non‐hallux valgus, or interventional studies with a non‐hallux valgus control group, using pre‐surgery data for individuals with hallux valgus. Eligible studies involved adult participants with and without hallux valgus deformity. Studies were included if they involved participants diagnosed with hallux valgus, as defined by the original study authors. Pediatric or adolescent populations were excluded unless explicitly identified as adults in the original studies.

The primary outcome measures included pressure, force, or their time integrals at the hallux and forefoot regions (medial and central areas) during walking gait. We excluded studies involving rheumatoid arthritis, diabetic foot, as well as cadaveric specimens, sawbone models, and computer simulations, in addition to those reporting a mixture of hallux valgus with other deformities.

### 2.2 Search Strategy and Selection Process

A systematic literature search was conducted on 29 July 2024, using PubMed, Web of Science, Scopus, CINAHL, and Embase. The search strategy employed a combination of keywords related to hallux valgus and plantar pressure measurement. The specific search terms used were as follows: [((foot OR plantar) AND (load* or pressure* or force* or impulse)) OR pedobarograph* OR barograph* OR baropodometr* OR pedograph*] AND [“hallux valgus” OR “hallux abducto valgus” OR “hallux abductovalgus” OR bunion OR “metatarsus primus varus”]. Detailed search terms, fields, and hits for each database are available in the supplementary materials (Tables S1 and S2).

The literature search encompassed English‐language journal articles from database inception to July 29, 2024. The first author conducted the initial search, followed by two independent reviewers (second and third authors) who screened titles, keywords, and abstracts. Subsequently, three reviewers assessed full‐text articles for eligibility. Any disagreements were resolved through discussion or consultation with additional authors.

### 2.3 Data Extraction and Synthesis

Data extraction was performed independently by two reviewers and summarized using a standardized form, including participant information, equipment modalities, and outcomes. Any discrepancies were resolved through discussion with the other authors.

For the meta‐analysis, we extracted plantar load (force and pressure) and plantar impulse (force‐time and pressure‐time integrals) data for the following regions: hallux, medial metatarsal, and central metatarsal. When studies reported individual metatarsal data, we defined the medial metatarsal region as the first metatarsal and the central metatarsal as the second and third metatarsals. Separate meta‐analyses were conducted for each of the metrics because some studies reported both. For studies presenting only graphical data without numeric values, we used GetData Graph Digitizer software to extract the required numeric data. If the data of a study lacked standard deviations (errors), they were imputed using the average of the standard deviations from other studies using the same metric. All extracted data were independently verified by another reviewer.

A frequentist meta‐analytic (random‐effects model) approach was employed using the standardized mean difference (SMD) to account for variations in measurement metrics across studies. Separate meta‐analyses were conducted for load and impulse in each foot region (hallux, medial metatarsal, and central metatarsal).

Heterogeneity was quantified using the I^2^ statistic, with values of 75% considered high heterogeneity [21]. We conducted subgroup analyses based on the measurement modality (platform system, in‐shoe sensor) and metrics (force, pressure). Publication bias tests through funnel plots and Egger's test were not conducted due to substantial heterogeneity or an insufficient number of studies in the meta‐analyses or subgroup analyses [21].

### 2.4 Risk‐of‐Bias Assessment

To assess the risk of bias in the included studies, we utilized the Joanna Briggs Institute (JBI) critical appraisal checklist for analytical cross‐sectional studies [22]. This tool consists of eight questions that evaluate study quality and potential sources of bias: (Q1) inclusion criteria, (Q2) study description, (Q3) exposure measurement, (Q4) outcome measurement, (Q5) confounder identification, (Q6) confounder management, (Q7) outcome reliability, and (Q8) statistical analysis.

## 3 Results

### 3.1 Study Selection

**FIGURE 2:**
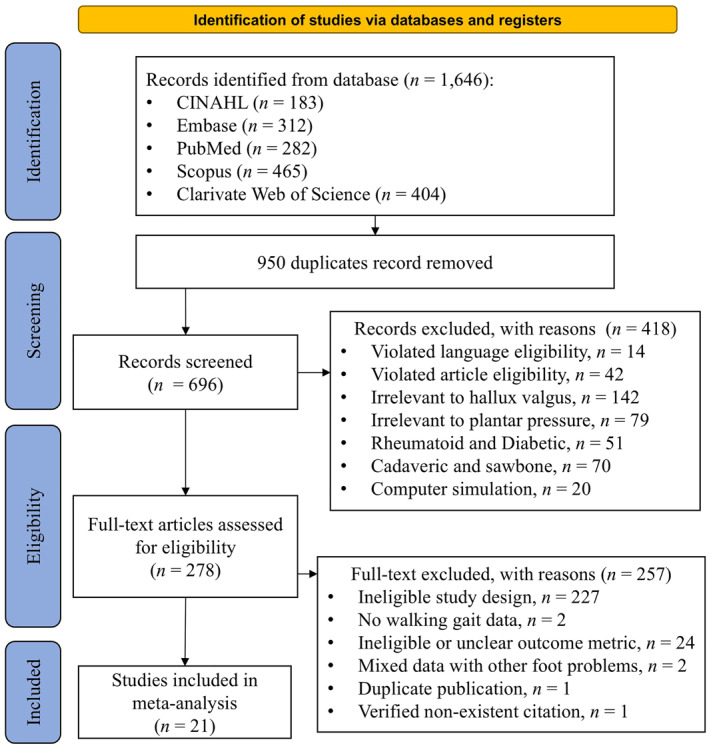
PRISMA flowchart of systematic search and study selection.

Figure 2 illustrates the PRISMA flowchart for the systematic search and screening process. The initial search yielded 1646 hits, from which 950 duplicates were removed. The primary screening of titles, abstracts, and keywords from the remaining 696 records identified 278 relevant studies. During this phase, 418 studies were excluded for various reasons: language requirement violations (*n* = 14), article‐type requirement violations (*n* = 42), unrelated to hallux valgus (*n* = 142), unrelated to plantar pressure (*n* = 79), focused on rheumatoid arthritis or diabetic foot (*n* = 51), involving cadaveric or sawbone specimens (*n* = 70), and computer simulations (*n* = 20).

Subsequent eligibility screening of the full texts further excluded 257 studies due to ineligible study design (*n* = 227), lack of walking gait data (*n* = 2), ineligible or unclear outcome metrics (*n* = 24), being mixed with other foot problems (*n* = 2), duplicate publications, and one verified nonexistent record from the journal. Ultimately, 21 articles were deemed eligible for the meta‐analysis [6, 17, 23, 24, 25, 26, 27, 28, 29, 30, 31, 32, 33, 34, 35, 36, 37, 38, 39, 40, 41].

### 3.2 Study Characteristics

The systematic review included 21 eligible studies, comprising a total of 3911 participants. Of these, 1336 individuals had hallux valgus (18% male, 77% female, 5% unspecified), whereas 2575 individuals did not (39% male, 58% female, 3% unspecified). The study population encompassed 1935 hallux valgus feet and 4472 non‐hallux valgus feet. It is important to note that the Framingham Foot Study by Galica et al. [6] contributed significantly to the overall sample size, accounting for 2414 participants. The remaining studies had considerably smaller sample sizes, ranging from 21 to 177 participants when combining both targeted and control groups. The representative age across studies ranged from 19 to 72 years for both groups. The pooled mean age was 50.4 years for the hallux valgus group and 47.2 years for the non‐hallux valgus group.

The included studies exhibited diverse designs and inclusion criteria. Six studies compared presurgical and postsurgical outcomes with normal control participants [25, 27, 31, 38, 39, 40]. Regarding hallux valgus severity, seven studies did not specify the required level of severity in their inclusion criteria [6, 17, 23, 24, 25, 38, 41]. Three studies focused on mild cases [29, 35, 36], one examined mild‐to‐moderate cases [34], and another investigated moderate cases [33]. Seven studies concentrated on moderate‐to‐severe cases [26, 27, 30, 31, 37, 39, 40]. Notably, two studies conducted stratified analyses on mild, moderate, and severe cases [28, 32].

The studies employed two primary modalities for data collection: platform‐based systems and in‐shoe sensors. Platform‐based systems were utilized in 10 studies, whereas in‐shoe sensors were employed in 11 studies. Among the studies using in‐shoe sensors, the methodologies varied considerably. Five studies adhered the sensors directly to the plantar surface of the feet and conducted experiments with participants barefoot [26, 30, 31, 39, 40]. Two studies had participants wear their own shoes with the sensors inserted [32, 34]. Another two studies provided participants with the same type of shoes for uniformity [35, 36]. Lastly, one study used the same shoes for all participants [38].

To conduct the meta‐analysis, we addressed several data issues across the included studies. Two studies did not report standard deviations for their outcome measures, requiring us to impute these values using established statistical methods. In two other studies, the relevant data were only presented in graphical format. For these, we employed data digitalization techniques to extract the necessary numerical values from the published charts.

### 3.3 Risk‐of‐Bias Assessment

**FIGURE 3:**
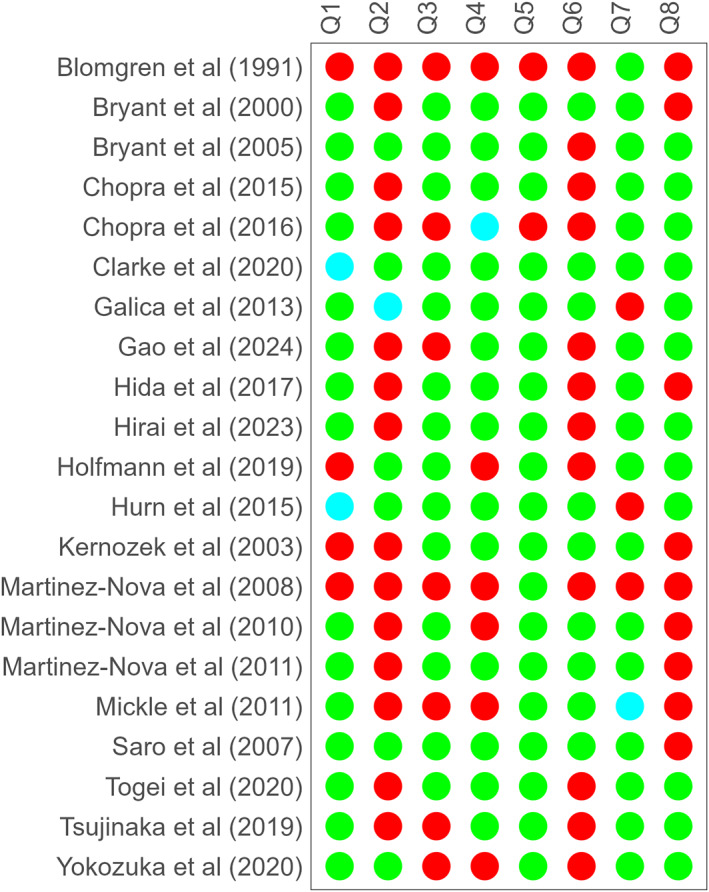
Risk‐of‐bias assessment using the Joanna Briggs Institute (JBI) critical appraisal checklist for analytical cross‐sectional studies on eight domains (Q1–Q8). Green: yes; red: no; cyan: unclear.

The median point of the risk‐of‐bias assessment was five out of eight, with an interquartile range of one. As shown in Figure 3, strengths were observed in confounder identification, with 91% of studies adequately addressing this domain. Similarly, “outcome reliability” was demonstrated in 81% of the studies. “Inclusion criteria” were clearly defined in 71% of the studies, whereas “exposure measurement” and “outcome measurement” were appropriately conducted in 67% of cases.

However, notable weaknesses were identified in several areas. “Study description” was inadequately addressed in about two‐thirds of the studies, which limited the reproducibility and interpretation of the results. “Confounder management” was insufficiently conducted in 57% of the studies, whereas 43% of the studies might not meet criteria for appropriate “statistical analysis.”

### 3.4 Meta‐Analysis

#### 3.4.1 Hallux Region

**FIGURE 4:**
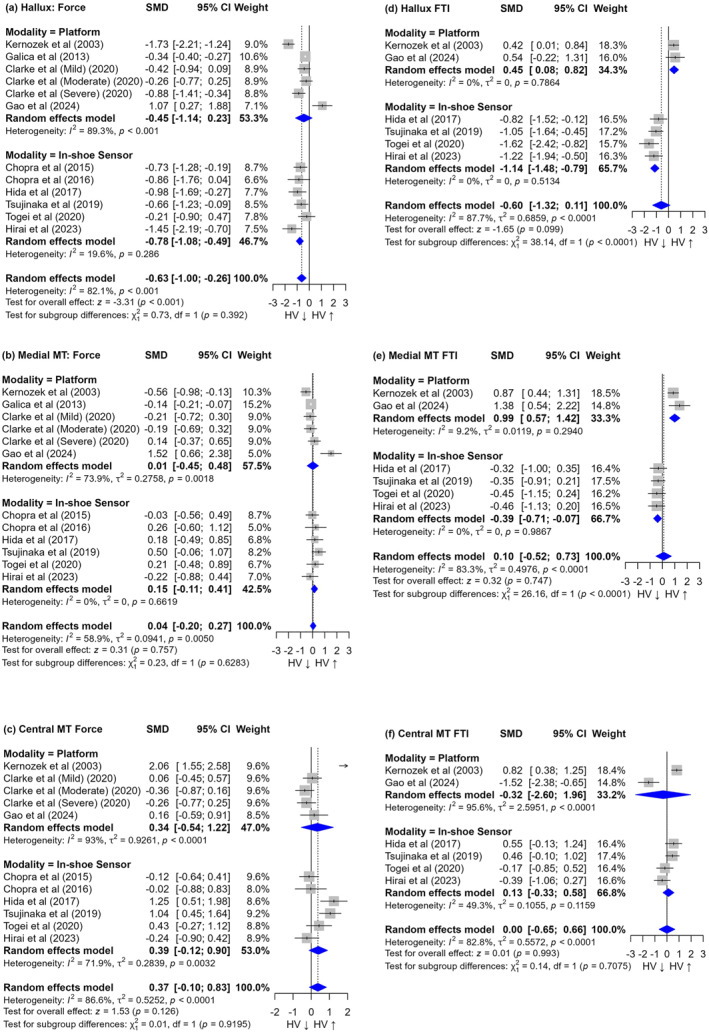
Forest plots of meta‐analyses on the force (a to c) and FTI (d to f) of the hallux, medial MT, and central MT regions. FTI: force‐time integral; MT: metatarsal.

As shown in Figure 4, in the hallux region, the overall effect size for force was −0.63 [95% CI: −1.00, 0.26], with high heterogeneity (I^2^ = 82.1%). Subgroup analysis by measurement modality partially resolved this heterogeneity, showing a significant reduction in force for in‐shoe measurements (SMD = −0.78 [95% CI: −1.08, −0.49], I^2^ = 19.6%), whereas platform measurements showed no significant difference (SMD = −0.45 [95% CI: −1.14, 0.23], I^2^ = 89.3%). For pressure in the hallux region (Figure S1), the overall effect size was −0.50 [95% CI: −1.22, 0.22], with very high heterogeneity (I^2^ = 95%). Subgroup analysis did not resolve the heterogeneity and revealed contradictory trends: Platform measurements showed a nonsignificant increase (SMD = 0.38 [95% CI: −0.02, 0.78], I^2^ = 94.6%), whereas in‐shoe measurements indicated a significant decrease (SMD = −1.70 [95% CI: −3.07, −0.32], I^2^ = 94.5%).

The force‐time integral analysis in the hallux region yielded an overall effect size of −0.60 [95% CI: −1.32, 0.11], with high heterogeneity (I^2^ = 87.7%). Subgroup analysis fully resolved the heterogeneity but revealed opposing directions: Platform measurements showed a significant increase (SMD = 0.45 [95% CI: 0.08, 0.82], I^2^ = 0%), whereas in‐shoe measurements showed a significant decrease (SMD = −1.14 [95% CI: −1.48, −0.79], I^2^ = 0%). The subgroup difference was statistically significant (*p* < 0.001), highlighting the contradictory results between measurement modalities. The pressure‐time integral in the hallux region (Figure S4), based solely on in‐shoe measurements, showed a significant large effect size of −3.45 [95% CI: −6.27, −0.62], with very high heterogeneity (I^2^ = 97.6%). This result aligns with the in‐shoe pressure and force‐time integral findings, suggesting a consistent decrease in load under the hallux for in‐shoe measurements.

#### 3.4.2 Medial Metatarsal Region

In the medial metatarsal region, the overall effect size for force was 0.04 [95% CI: −0.20, 0.27], with moderate heterogeneity (I^2^ = 58.9%), as shown in Figure 4. Subgroup analysis partially resolved the heterogeneity for in‐shoe measurements (I^2^ = 0%) but not for platform measurements (I^2^ = 73.9%). No significant differences were found between platform and in‐shoe measurements, indicating consistency across modalities. For pressure in the medial metatarsal region (Figure S2), the overall effect size was −0.08 [95% CI: −0.44, 0.29], with high heterogeneity (I^2^ = 89.6%). Subgroup analysis did not resolve the heterogeneity and revealed no significant differences for either platform or in‐shoe measurements, suggesting agreement between modalities despite high variability.

The force‐time integral analysis in the medial metatarsal region showed an overall effect size of 0.10 [95% CI: −0.52, 0.73], with high heterogeneity (I^2^ = 83.3%). Subgroup analysis resolved the heterogeneity but revealed contradictory results: a significant increase for platform measurements (SMD = 0.99 [95% CI: 0.57, 1.42], I^2^ = 9.2%) and a nonsignificant decrease for in‐shoe measurements (SMD = −0.39 [95% CI: −0.71, 0.07], I^2^ = 0%). The subgroup difference was statistically significant (*p* < 0.001), highlighting the impact of measurement modality on results. The pressure‐time integral in the medial metatarsal region (Figure S5), based on in‐shoe measurements only, showed a nonsignificant effect size of −1.45 [95% CI: −3.18, 0.28], with very high heterogeneity (I^2^ = 96.6%). This result aligns with the direction of the in‐shoe force‐time integral findings, although the magnitude differs.

#### 3.4.3 Central Metatarsal Region

In the central metatarsal region, the overall effect size for force was 0.37 [95% CI: −1.00, 0.83], with high heterogeneity (I^2^ = 86.6%), as shown in Figure 4. Subgroup analysis did not substantially resolve the heterogeneity and showed no significant differences between platform and in‐shoe measurements, suggesting consistency across modalities despite high variability. For pressure in the central metatarsal region (Figure S3), the overall effect size was 0.09 [95% CI: −0.26, 0.44], with high heterogeneity (I^2^ = 89.7%). Subgroup analysis did not resolve the heterogeneity and revealed no significant differences for either platform or in‐shoe measurements, indicating agreement between modalities.

The force‐time integral analysis in the central metatarsal region showed an overall effect size of 0.00 [95% CI: −0.65, 0.66], with high heterogeneity (I^2^ = 82.8%). Subgroup analysis partially resolved the heterogeneity for in‐shoe measurements (I^2^ = 49.3%) but revealed no significant differences between platform and in‐shoe measurements, suggesting consistency across modalities. The pressure‐time integral in the central metatarsal region (Figure S6), based on in‐shoe measurements only, showed a nonsignificant effect size of −1.05 [95% CI: −2.29, 0.19], with high heterogeneity (I^2^ = 93.8%). This result somewhat contradicts the direction of the other measures in this region, which showed slight positive trends, although nonsignificant.

## 4 Discussion

The meta‐analyses of plantar biomechanics in individuals with hallux valgus, compared to normal controls, revealed several key findings that partially support our initial hypotheses. In the hallux region, we observed a consistent trend of reduced loading in individuals with hallux valgus, particularly for in‐shoe measurements. This was evidenced by overall decreases in force, pressure, force‐time integral, and pressure‐time integral when measured in‐shoe. These findings aligned with our hypothesis of reduced load‐bearing capacity in the hallux region due to the structural deformity associated with hallux valgus. Focusing on the meta‐analysis with low heterogeneity, we could conclusively determine that, using in‐shoe sensors, the force under the hallux is significantly reduced (SMD: −0.89 [95% CI: −1.08, −0.49]). Additionally, the impulse under the hallux showed a significant decrease (SMD: −0.39 [95% CI: −0.71, −0.07]). Reduced hallux loading indicates the need for targeted interventions to restore medial column stability and enhance gait mechanics. Physiotherapy should emphasize strengthening intrinsic foot muscles and incorporating balance training to mitigate lateral weight shifts [42, 43, 44]. Postoperative retraining is also crucial to strengthen muscles, optimize plantar pressure redistribution, and prevent recurrence [45, 46].

Contrary to our expectations, no conclusive evidence emerged for reduced medial metatarsal loading. Although some studies associate decreased pressures with pain‐mediated avoidance strategies or mechanical insufficiency due to hallux pronation and sesamoid subluxation [6, 28, 36], the majority of studies demonstrated no significant changes. Notably, there were correlations between lower plantar loading, greater angular deformity, and sesamoid malposition [32, 47]. Additionally, Sanders et al. [48] reported an inverse relationship between the hallux valgus angle and hallux plantar flexion strength, particularly in symptomatic individuals. However, in mild cases, intact function may persist below pain and functional decline thresholds. We postulate that early‐stage cases might undergo a transitional compensatory phase, where the neuromuscular system increases forefoot loading to preserve push‐off kinematics and dynamic balance. Additionally, concurrent pes planus could contribute to increased load due to pronatory medial weight redistribution [49]. This suggested that the load transfer from the hallux to the medial metatarsals may be more complex than initially hypothesized.

Similarly, our hypothesis of increased loading in the central metatarsal region as a compensatory mechanism was not strongly supported by the data. Although slight positive trends were observed, high heterogeneity and nonsignificant results suggest complex interindividual variability in load redistribution mechanisms. Although not all patients with HV might develop metatarsalgia, the literature revealed divergent findings regarding central metatarsal loading patterns. Studies reporting decreased loading in the central metatarsal region attributed this reduction to foot pronation [41], whereas asymptomatic individuals may be protected through intact neuromuscular compensatory mechanisms stabilizing the medial column [50] and/or preserved transverse arch integrity [7], both of which appear to buffer against central metatarsal overload that can lead to metatarsalgia.

Moreover, the temporal dimension of mechanical loading likely plays a crucial role, as calluses represent the cumulative effect of repetitive load over extended periods [51]. There is no information on how long patients have had hallux valgus or how long the altered loading patterns have been present before resulting in callus formation. Furthermore, conventional plantar pressure measurement systems primarily quantify vertical forces (or pressures) while potentially overlooking the contribution of shear forces (or friction) to tissue stress [51]. Research has demonstrated that plantar shear experienced by patients with calluses during the push‐off phase is significantly higher [52]. Although some researchers have attempted to address this by computer simulation [53] or installations of specialized shear stress transducers [54, 55], particularly within high‐heeled footwear, the accurate measurement or estimation of plantar shear remains challenging with standard pressure measurement systems.

Identifying the most suitable methods and outcome metrics for assessing foot and ankle conditions and their treatments is considered a top research priority in foot and ankle surgery [56]. Clinically, first ray insufficiency has traditionally been assessed using manual examination techniques, such as the Klaue device [57], which directly quantifies first ray hypermobility through dorsal‐plantar displacement measurements and primarily evaluates sagittal plane mobility in non‐weight‐bearing positions, providing static measurement of structural instability. However, these methods cannot capture the dynamic functional consequences that occur during actual weight‐bearing activities and therefore may not reflect the multiplanar instability present in hallux valgus.

Plantar pressure measurement offers advantages for evaluating first ray insufficiency or regional load‐carrying capacity during functional activities. A previous study using radiokinematic and pedobarographic analysis demonstrated the relationship between first ray insufficiency and plantar pressure distribution [8]. The study revealed that instability of the first tarsometatarsal joint leads to unloading of the first metatarsal and a subsequent transfer of force to the central forefoot [8]. Different plantar pressure parameters offer complementary insights. Peak pressure represents the maximum force per unit area experienced at a specific location during gait. In hallux valgus assessment, these pressure values have dual significance based on anatomical location—elevated pressure under the first metatarsal typically indicates functional load‐bearing capacity, whereas increased pressure under the lesser metatarsals may signal pain (i.e., transfer metatarsalgia). Force‐time and pressure‐time integrals incorporate both magnitude and temporal characteristics of loading, better reflecting sustained mechanical stress throughout the stance phase, whereas proportional measurement using percentage of total foot normalized for individual weight and gait difference could enable more meaningful clinical comparisons. Some researchers have also employed center‐of‐pressure trajectory and transformation analysis [58, 59], which provided deeper insights into weight‐shifting mechanisms in hallux valgus that warrant a dedicated review and analysis.

Although an existing review suggested that hallux valgus surgeries may worsen plantar loading [4], our meta‐analysis found that the choice of measurement modality, specifically the use of platform plate (or pressure mat) systems versus in‐shoe sensors, emerged as a significant driver of heterogeneity in the estimates. This was evidenced by the resolution, either total or partial, of heterogeneity when subgrouping by modality, with significant subgroup differences observed. Notably, a Simpson's paradox was identified in the force‐time integral measurements for the hallux and medial metatarsal regions, where the effects appeared to be opposite between platform‐based systems and in‐shoe sensors. The observed modality‐dependent effects likely stem from fundamental differences in measurement context. Platform systems measure barefoot mechanics where hallux malalignment directly impacts ground contact patterns, whereas in‐shoe measurements reflect real‐world footwear constraints that mechanically restrict forefoot splaying through shoe upper counterpressure and alter load distribution via sole stiffness and toe box geometry [60]. These shod conditions may also induce compensatory strategies through orthotic use or footwear modifications [61, 62]. This discrepancy underscores the potential impact of shoe fit and shoe–foot interactions on plantar pressure distributions in individuals with hallux valgus. Although some studies have suggested that plate systems and in‐shoe sensors yield different results and should not be used interchangeably [63], our findings indicate that this disparity may be even more pronounced for individuals with foot deformities, potentially affecting clinical interpretation. The altered foot structure in patients with hallux valgus likely interacts differently with footwear, which may explain the observed differences between measurement modalities.

The heterogeneity observed in our meta‐analysis can be further attributed to several factors. Pain‐mediated load redistribution represents a critical confounding bias. Patients with painful hallux valgus may adopt compensatory gait patterns (e.g., lateral weight shifting, shortened stride length) that artificially reduce hallux loading independent of anatomical deformity severity [6, 28, 64]. Severe hallux valgus accompanied by hallux pain is believed to substantially reduce loading under the hallux [28]. A pain avoidance strategy may be demonstrated by a linear trend showing a reduction in the pressure‐time integral as hallux valgus severity increases [28, 32]. Symptomatic patients might increase reliance on the lateral forefoot or lesser toes. Secondly, inconsistent severity thresholds introduced spectrum bias. In particular, studies varied in their inclusion criteria, ranging from those that merely indicated the presence of hallux valgus to others focusing on mild cases or severe cases requiring surgical intervention. Given the variations in the criteria or severity classification among the reviewed articles, a meta‐regression analyzing the relationship between plantar pressures and hallux valgus severity would be valuable for future research.

Additionally, factors such as hypermobility, potentially induced by generalized ligament laxity, could be a source of hallux valgus and might vary among individuals or between genders, affecting the results [65, 66, 67]. Generalized ligament laxity has been implicated in the progression of hallux valgus by reducing joint stability and increasing deforming forces at the first metatarsophalangeal joint [13]. The co‐occurrence of other foot deformities, which are difficult to isolate, such as lesser toe deformities, foot pronation, or flatfoot, may also contribute to the high heterogeneity observed in plantar pressure measurements [68, 69]. These deformities may lead to additional compensatory mechanisms, such as increased midfoot or rearfoot loading [37, 70].

Study design issues, particularly related to control group selection, may also contribute to the observed heterogeneity. Nonoptimally matched controls and variations in sampling sources across studies can introduce bias and increase variability in the results. The risk‐of‐bias assessment revealed both strengths and limitations across the included studies. A notable strength was the identification and acknowledgment of potential confounders, demonstrating researchers' awareness of factors that could influence study outcomes. The majority of studies employed standardized protocols with multiple trials, enhancing the reliability of their findings. Furthermore, most studies provided clear inclusion criteria and diagnostic standards for hallux valgus, often utilizing a combination of radiographic evidence, clinical scores, and physical examinations. However, several methodological challenges were evident. Despite the identification of confounders, their management was often inadequate due to clinical setting constraints. Some researchers attempted to mitigate this issue through age‐matching control groups or stratifying analyses by hallux valgus severity. A common weakness was the lack of clear study descriptions, particularly regarding sampling sources and approaches. Additionally, the statistical rigor of some studies was compromised by the absence of data normality assessments or outlier identification prior to applying parametric tests.

There were some limitations in this review. Firstly, our search was limited to English, potentially excluding relevant studies published in other languages and introducing language or selection bias. Secondly, due to the high heterogeneity observed, we were unable to conduct a formal assessment of publication bias. The substantial heterogeneity also limited the generalizability of our findings and allowed us to draw only limited conclusions. One limitation of this review is the lack of strict constraints on the age range and diagnostic methods for hallux valgus across the included studies. By relying on the original studies' definitions of “adults” without imposing a specific age threshold, there may be variability in the age distribution of participants, potentially including younger adults or older adolescents categorized as adults. Older adults experience significant changes in foot morphology, biomechanics, and topology compared to younger adults [71, 72]. Additionally, we did not restrict inclusion based on the method of hallux valgus diagnosis. Although this approach ensured inclusivity, it may have introduced heterogeneity in how hallux valgus was identified and classified across studies, potentially influencing the pooled outcomes. Furthermore, methodological limitations in pain documentation and severity stratification were impactful. However, we did not conduct meta‐regression or subgroup analyses based on pain level and hallux valgus severity. This decision was made because some studies pooled multiple severity groups and employed varying definitions of severity levels, whereas others did not have the information (e.g., hallux valgus angle) available.

## 5 Conclusion

This meta‐analysis provides valuable insights into plantar pressure distribution patterns in individuals with hallux valgus, highlighting both consistent findings and areas of variability. Reduced loading under the hallux was observed predominantly in studies utilizing in‐shoe sensors, confirming diminished hallux pressure as a biomechanical consequence of hallux valgus. However, compensatory increases in loading under the medial and central metatarsals, anticipated based on biomechanical theories, were not consistently supported across studies. This inconsistency underscores the significant heterogeneity in study methodologies and populations.

The high heterogeneity observed in this analysis stems from several factors, including differences in measurement modalities, sample characteristics (e.g., age, gender), disease severity, and statistical approaches. Notably, platform‐based systems and in‐shoe sensors yielded divergent results due to fundamental differences in measurement contexts—barefoot mechanics versus shod conditions—which influence plantar load redistribution. Pain‐mediated gait adaptations and coexisting foot deformities (e.g., flatfoot or lesser toe deformities) further contribute to variability, as do inconsistent severity thresholds and confounder management across studies.

This heterogeneity has important implications for interpreting study results. Although subgroup analyses partially resolved some sources of variability, the robustness of findings remains limited, particularly for medial and central metatarsal loading. Clinicians should interpret these findings with caution, recognizing that methodological inconsistencies may impact generalizability and clinical applicability.

Future research should prioritize standardizing measurement protocols and phenotyping HV factors, such as hallux valgus severity, pain levels, and coexisting deformities. Additionally, meta‐regression analyses exploring the relationship between plantar pressure patterns and disease severity could provide deeper insights into compensatory mechanisms. Such efforts will enhance the reliability of plantar pressure assessments and their utility in guiding clinical decision‐making for hallux valgus management.

## Author Contributions

Conceptualization: J.C.W.C., M.N., A.K.L.L. Methodology: J.C.W.C., M.N., A.K.L.L. Formal analysis: D.W.C.W., E.M.W.C., L.L.L., J.W., T.C.T.M. Investigation: D.W.C.W., E.M.W.C., L.L.L., J.W., T.C.T.M. Data curation: D.W.C.W., E.M.W.C., L.L.L. Validation: E.M.W.C., L.L.L., J.W., T.C.T.M. Supervision: J.C.W.C., M.N., A.K.L.L. Funding acquisition: M.N. Writing – original draft preparation: D.W.C.W., E.M.W.C., L.L.L. Writing – review and editing: J.C.W.C., M.N., A.K.L.L.

## Ethics Statement

The authors have nothing to report.

## Consent

The authors have nothing to report.

## Conflicts of Interest

The authors declare no conflicts of interest.

## Supporting information


**Figure S1**: Forest plot of meta‐analysis on the pressure of the hallux region.

**Figure S2**: Forest plot of meta‐analysis on the pressure of the medial metatarsal region.

**Figure S3**: Forest plot of meta‐analysis on the pressure of the central metatarsal region.

**Figure S4**: Forest plot of meta‐analysis on the pressure‐time integral of the hallux region.

**Figure S5**: Forest plot of meta‐analysis on the pressure‐time integral of the medial metatarsal region.

**Figure S6**: Forest plot of meta‐analysis on the pressure‐time integral of the central metatarsal region.

**Table S1**: Full search terms and queries for the systematic database search.

**Table S2**: Search queries, hits, and entries from various databases (search date: 29 July 2024).

## Data Availability

Data sharing is not applicable to this review article as no new data were created or analysed in this study.
