# Supplementary figures and images for: Does Hallux Valgus Impair Medial Forefoot Loading? A Meta‐Analysis of Plantar Pressure Distribution
Source: J Foot Ankle Res. 2025 Aug 11;18(3):e70073. doi: 10.1002/jfa2.70073 (PMC12339410; doi:10.1002/jfa2.70073)

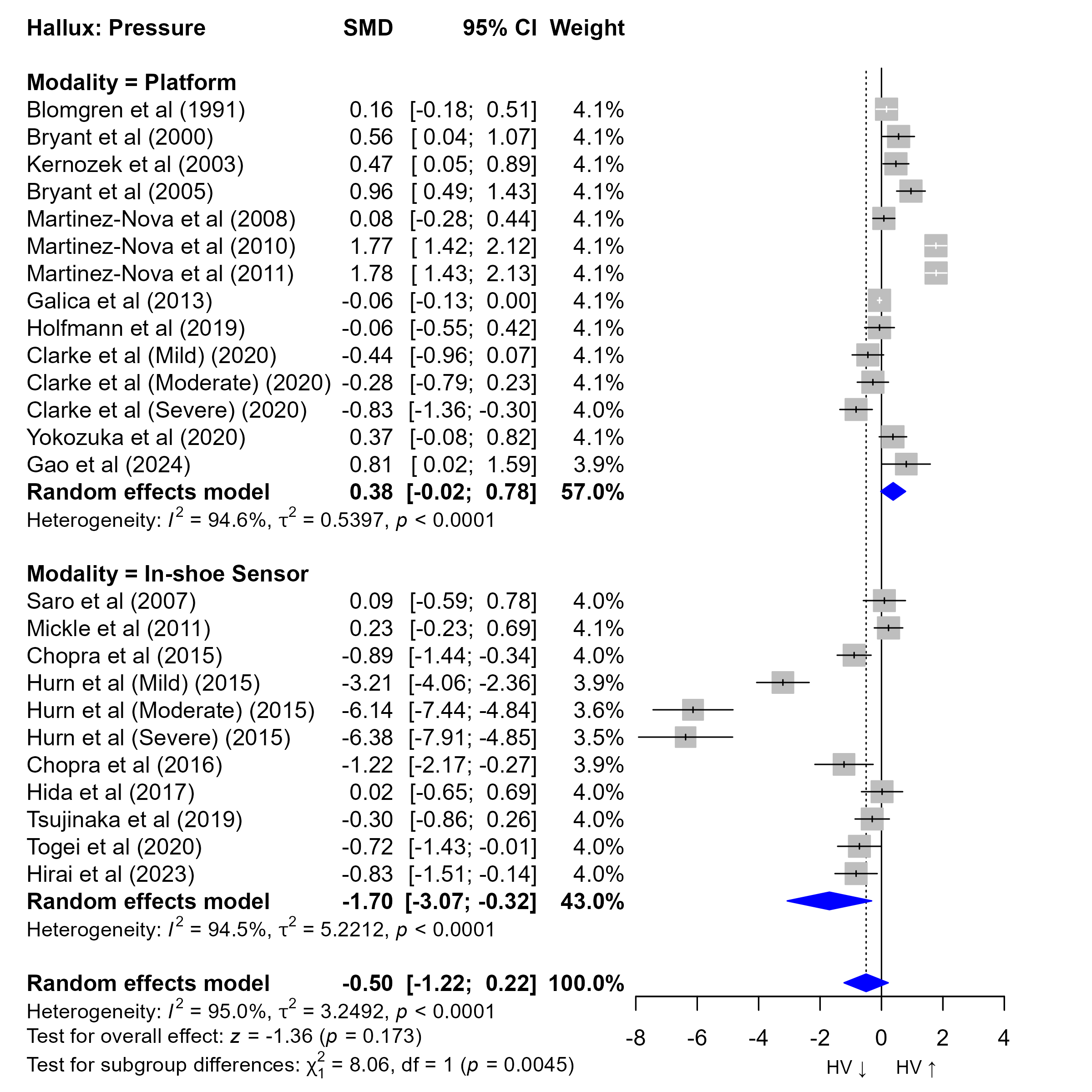

Supplement: Supplementary file 1 — Figure S1: Forest plot of meta‐analysis on the pressure of the hallux region. [file JFA2-18-e70073-s006.png]

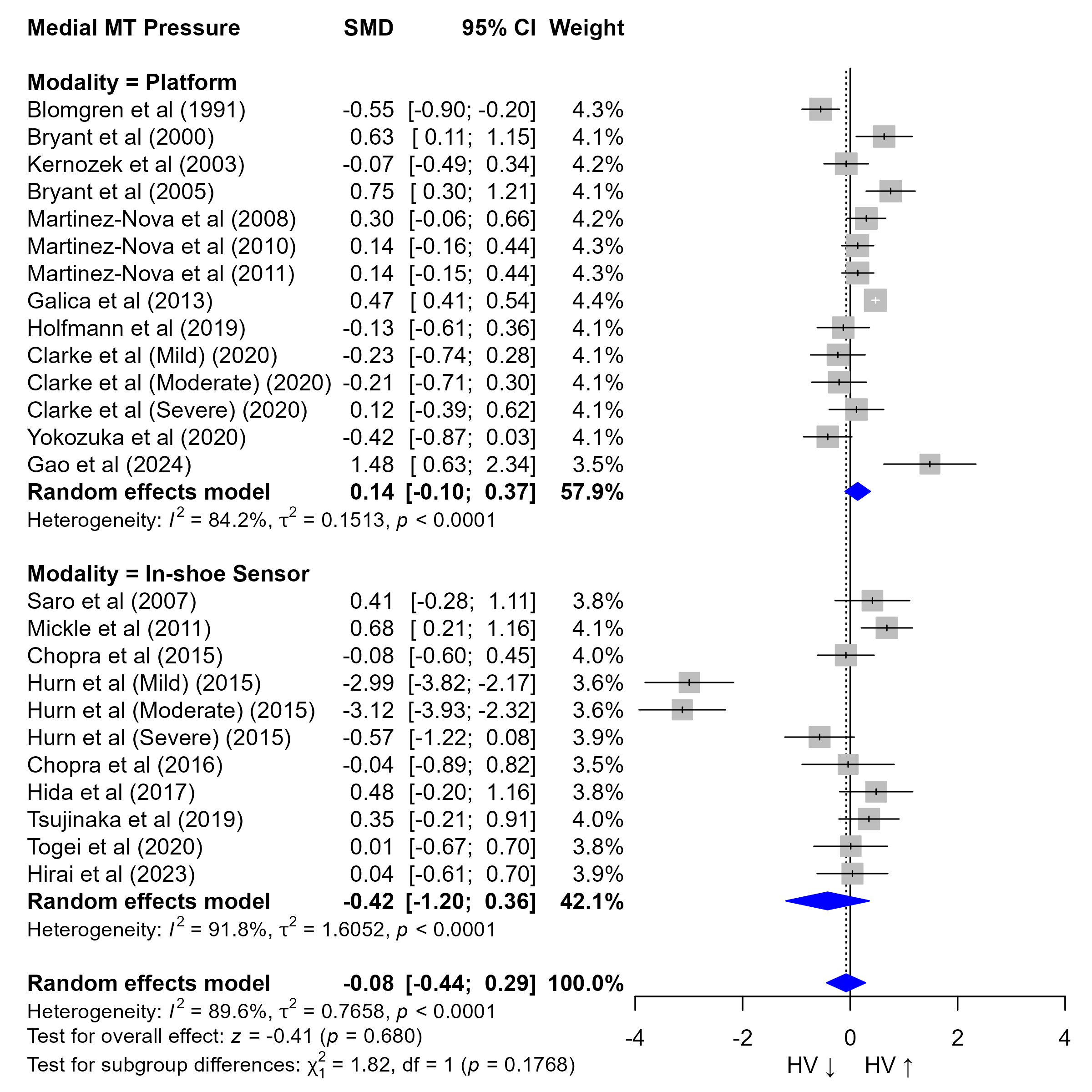

Supplement: Supplementary file 2 — Figure S2: Forest plot of meta‐analysis on the pressure of the medial metatarsal region. [file JFA2-18-e70073-s007.png]

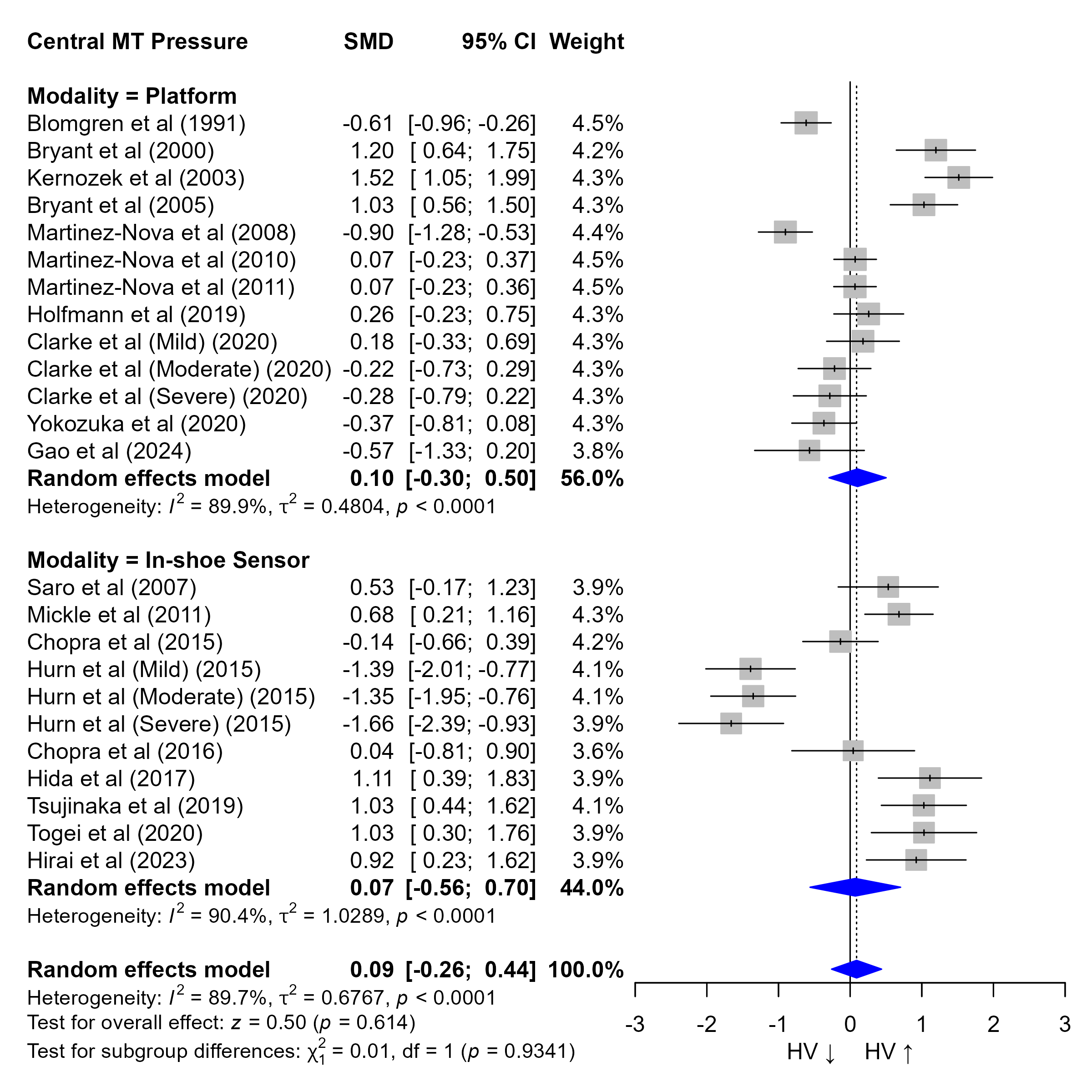

Supplement: Supplementary file 3 — Figure S3: Forest plot of meta‐analysis on the pressure of the central metatarsal region. [file JFA2-18-e70073-s003.png]

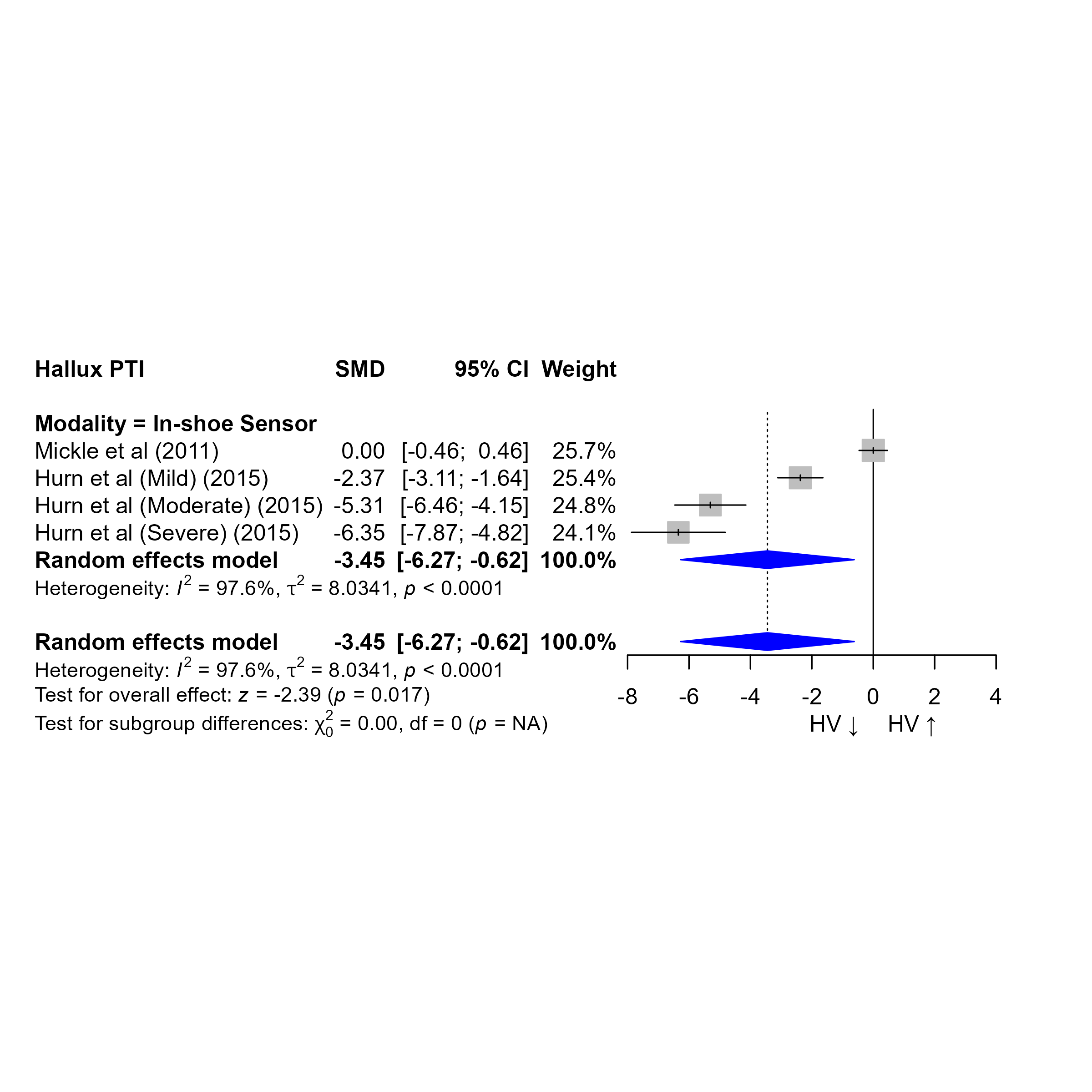

Supplement: Supplementary file 4 — Figure S4: Forest plot of meta‐analysis on the pressure‐time integral of the hallux region. [file JFA2-18-e70073-s001.png]

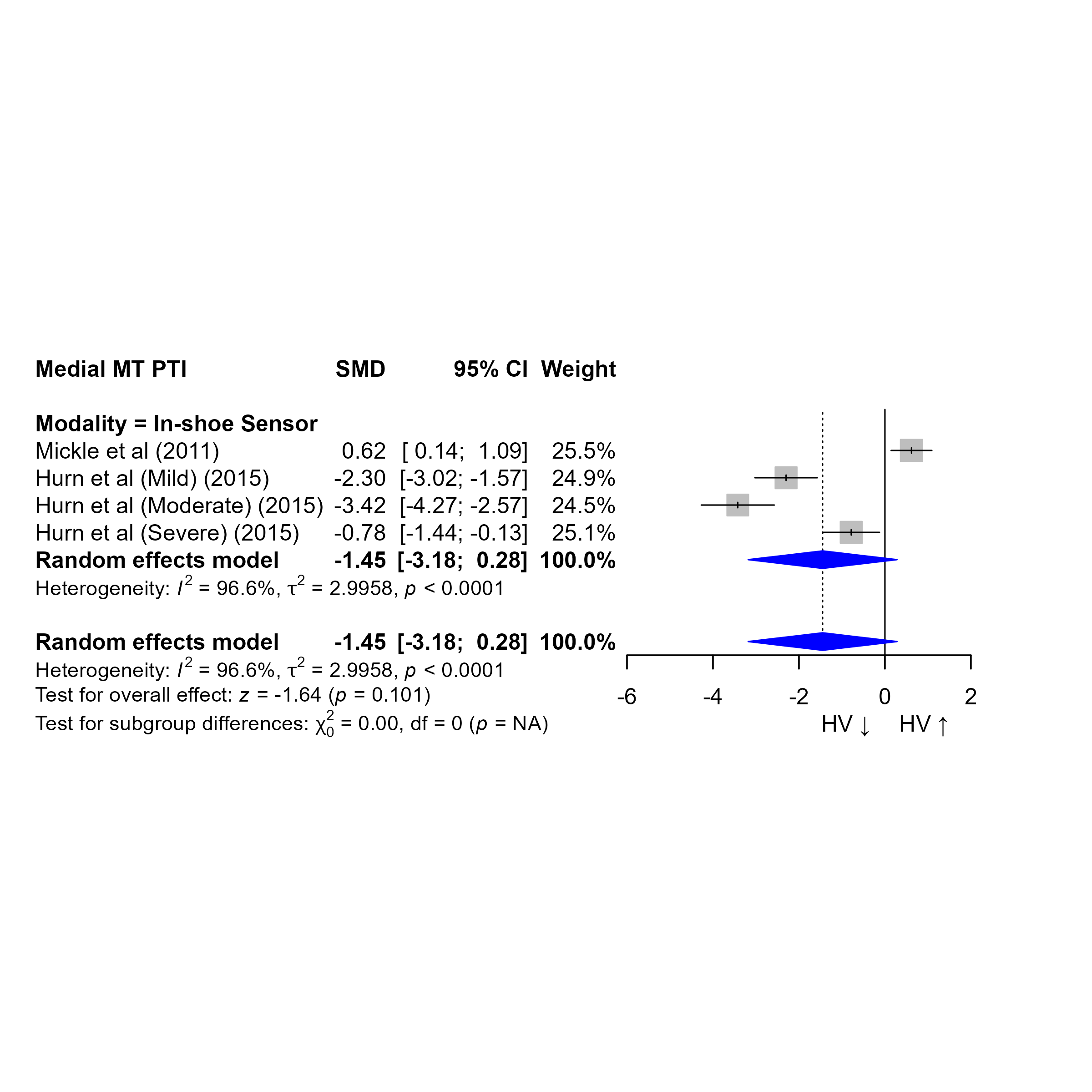

Supplement: Supplementary file 5 — Figure S5: Forest plot of meta‐analysis on the pressure‐time integral of the medial metatarsal region. [file JFA2-18-e70073-s008.png]

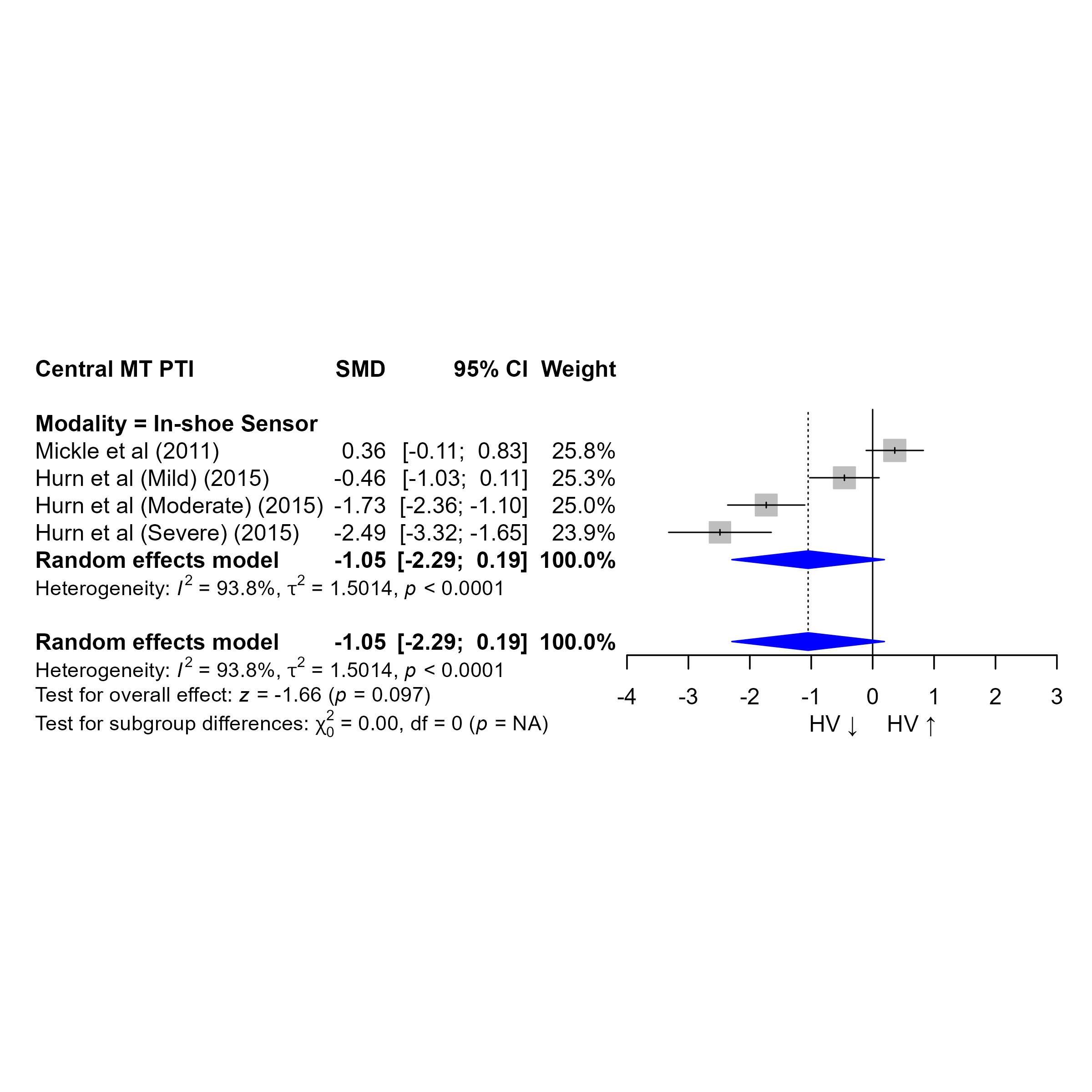

Supplement: Supplementary file 6 — Figure S6: Forest plot of meta‐analysis on the pressure‐time integral of the central metatarsal region. [file JFA2-18-e70073-s005.png]
