# Supplementary material for: Does Hallux Valgus Impair Medial Forefoot Loading? A Meta‐Analysis of Plantar Pressure Distribution
Source: J Foot Ankle Res. 2025 Aug 11;18(3):e70073. doi: 10.1002/jfa2.70073 (PMC12339410; doi:10.1002/jfa2.70073)
Supplement: Supplementary file 7 — Table S1: Full search terms and queries for the systematic database search. [file JFA2-18-e70073-s002.docx]

**Table S1**. Full search terms and queries for the systematic database search.

|  | Domain | Terms |
| --- | --- | --- |
| #1 | Participants | ((Foot OR plantar) AND (Load* or Pressure* or Force* or Impulse)) OR pedobarograph* OR barograph* OR baropodometr* OR pedograph* |
| #2 | Exposure | "hallux valgus" OR "hallux abducto valgus" OR "hallux abductovalgus" OR bunion OR "metatarsus primus varus" |

(#1 AND #2), Filter: English, Date: from inception.
