# Supplementary material for: Does Hallux Valgus Impair Medial Forefoot Loading? A Meta‐Analysis of Plantar Pressure Distribution
Source: J Foot Ankle Res. 2025 Aug 11;18(3):e70073. doi: 10.1002/jfa2.70073 (PMC12339410; doi:10.1002/jfa2.70073)
Supplement: Supplementary file 8 — Table S2: Search queries, hits, and entries from various databases (search date: 29 July 2024). [file JFA2-18-e70073-s004.docx]

**Table S2.** Search queries, hits, and entries from various databases (Search Date: 29 Jul 2024)

| Search Queries for PubMed | | |
| --- | --- | --- |
| **Number** | **Search Terms** | **Search Hits** |
| #1 | (("Foot"[Title/Abstract] OR "plantar"[Title/Abstract]) AND ("Load"[Title/Abstract] OR "Pressure"[Title/Abstract] OR "Force"[Title/Abstract])) OR "pedobarograph*"[Title/Abstract] OR "barograph*"[Title/Abstract] OR "baropodometr*"[Title/Abstract] OR "pedograph*"[Title/Abstract] | 18,154 |
| #2 | "hallux valgus"[Title/Abstract] OR "hallux abducto valgus"[Title/Abstract] OR "hallux abductovalgus"[Title/Abstract] OR "bunion"[Title/Abstract] OR "metatarsus primus varus"[Title/Abstract] | 4,393 |
| #3 | #1 AND #2 | 312 |
| #4 | #3 Filters: English | 282 |
| Search Queries for Web of Science | | |
| **Number** | **Search Terms** | **Search Hits** |
| #1 | TS=(((Foot OR plantar) AND (Load or Pressure or Force)) OR pedobarograph* OR barograph* OR baropodometr* OR pedograph*) | 39,803 |
| #2 | TS=("hallux valgus" OR "hallux abducto valgus" OR "hallux abductovalgus" OR bunion OR "metatarsus primus varus" ) | 3,789 |
| #3 | #1 AND #2 | 485 |
| #4 | #3 and Article (Document Types) and English (Languages) | 404 |
| Search Queries for Scopus | | |
| **Number** | **Search Terms** | **Search Hits** |
| #1 | TITLE-ABS-KEY ( ( ( foot OR plantar ) AND ( load OR pressure OR force ) ) OR pedobarograph* OR barograph* OR baropodometr* OR pedograph* ) | 53,385 |
| #2 | TITLE-ABS-KEY ( "hallux valgus" OR "hallux abducto valgus" OR "hallux abductovalgus" OR bunion OR "metatarsus primus varus" ) | 7,565 |
| #3 | #1 AND #2 | 655 |
| #4 | #3 AND (LIMIT-TO (DOCTYPE, “ar”) AND (LIMIT-TO (LANGUAGE, “English”)) | 465 |
| Search Queries for CINAHL via EbscoHost | | |
| **Number** | **Search Terms** | **Search Hits** |
| S1 | ((Foot OR plantar) AND (Load or Pressure or Force)) OR pedobarograph* OR barograph* OR baropodometr* OR pedograph* | 9,557 |
| S2 | "hallux valgus" OR "hallux abducto valgus" OR "hallux abductovalgus" OR bunion OR "metatarsus primus varus" | 2,857 |
| S3 | S1 AND S2 | 184 |
| S4 | S3, Narrow by Language - English | 183 |
| Search Queries for Embase | | |
| **Number** | **Search Terms** | **Search Hits** |
| #1 | (( ( Foot OR plantar ) AND ( Load or Pressure or Force ) ) OR pedobarograph* OR barograph* OR baropodometr* OR pedograph*):ti,ab,kw | 24,419 |
| #2 | ("hallux valgus" OR "hallux abducto valgus" OR "hallux abductovalgus" OR bunion OR "metatarsus primus varus"):ti,ab,kw | 5,531 |
| #3 | #1 AND #2 | 412 |
| #4 | #3 AND ‘article’/it | 312 |
